# Detection of *Wuchereria bancrofti* L3 Larvae in Mosquitoes: A Reverse Transcriptase PCR Assay Evaluating Infection and Infectivity

**DOI:** 10.1371/journal.pntd.0000602

**Published:** 2010-02-16

**Authors:** Sandra J. Laney, Reda M. R. Ramzy, Hanan H. Helmy, Hoda A. Farid, Ameen A. Ashour, Gary J. Weil, Steven A. Williams

**Affiliations:** 1 Department of Biological Sciences, Smith College, Northampton, Massachusetts, United States of America; 2 Zoology Department, Ain Shams University, Cairo, Egypt; 3 National Nutrition Institute, Cairo, Egypt; 4 Research and Training Center on Vectors of Diseases, Ain Shams University, Cairo, Egypt; 5 Department of Entomology, Ain Shams University, Cairo, Egypt; 6 Faculty of Science, Taif University, Taif, Kingdom of Saudi Arabia; 7 Infectious Disease Division, Department of Internal Medicine, Washington University School of Medicine, St. Louis, Missouri, United States of America; 8 Molecular and Cellular Biology, University of Massachusetts, Amherst, Massachusetts, United States of America; University of Pittsburgh, United States of America

## Abstract

**Background:**

Detection of filarial DNA in mosquitoes by PCR cannot differentiate infective mosquitoes from infected mosquitoes. In order to evaluate transmission risk an assay is needed that can specifically detect infective L3 stage parasites. We now report the development of an assay that specifically detects the infective stage of *Wuchereria bancrofti* in mosquitoes. The assay detects an L3-activated mRNA transcript by reverse-transcriptase PCR (RT-PCR).

**Methodology/Principal Findings:**

*W. bancrofti* cuticle-related genes were selected using bioinformatics and screened as potential diagnostic target genes for L3 detection in mosquitoes. Expression profiles were determined using RT-PCR on RNA isolated from mosquitoes collected daily across a two-week period after feeding on infected blood. Conventional multiplex RT-PCR and real-time multiplex RT-PCR assays were developed using an L3-activated cuticlin transcript for L3 detection and a constitutively expressed transcript, *tph-1*, for ‘any-stage’ detection.

**Conclusions/Significance:**

This assay can be used to simultaneously detect *W. bancrofti* infective stage larvae and ‘any-stage’ larvae in pooled vector mosquitoes. This test may be useful as a tool for assessing changes in transmission potential in the context of filariasis elimination programs.

## Author Summary

Lymphatic filariasis is a disabling and disfiguring disease caused by a parasite that is transmitted by a mosquito. The life cycle of the parasite requires two hosts: the mosquito vector and the human host. Part of the developmental life cycle of the parasite occurs in the mosquito and the other part in the human host. The parasite develops through four stages in the mosquito, only the last of which is infectious to humans. The third larval stage (L3) is the infective stage that initiates human infections when infective mosquitoes bite humans. There is currently a global program attempting to eliminate this disease by administering drugs to affected communities with the goal of interrupting transmission of the parasite. The new diagnostic tool described in this paper uses molecular techniques to specifically detect the infective stage of the parasite in mosquitoes. Many mosquitoes can be tested at one time to assess the risk of ongoing transmission of filariasis in communities. In addition, this new L3-detection assay can simultaneously detect whether the mosquitoes contain ‘any-stage’ of the parasite. This provides information on infection rates in humans in the community. Both pieces of information can be used in assessing the progress of disease elimination efforts.

## Introduction

Lymphatic Filariasis (LF) is a disabling, disfiguring, and poverty promoting disease that affects an estimated 120 million individuals in developing countries [1],[2]. The nematode parasite *Wuchereria bancrofti* is responsible for 90% of this global disease burden. This mosquito-borne disease threatens more than 1.2 billion individuals living in endemic countries [3]. For this reason, the Global Program to Eliminate Lymphatic Filariasis (GPELF) was established with the goal of eliminating LF as a public health problem by 2020 [4],[5]. The strategy for the interruption of disease transmission is largely based on mass drug administration (MDA) of antifilarial medications to endemic populations to treat those who are currently infected and to reduce the reservoir of parasites available to mosquitoes that transmit the infection.

An estimated 570 million people were treated between 2000 and 2007 in 48 countries using this yearly MDA strategy [3]. Currently, assessments of the success of the GPELF program are largely based on testing human blood to evaluate the infection status of affected communities [6],[7],[8]. PCR detection of parasites in mosquitoes, termed molecular xenomonitoring (MX) [9],[10], has also been used for monitoring the progress of elimination programs [7],[11],[12],[13],[14],[15]. Mosquito PCR provides an indirect measure of filarial infection rates in human populations, but is not a measure of transmission. This is because PCR detects DNA from all parasite stages in mosquitoes without distinction and therefore measures “infection” in mosquitoes and not “infectivity” as not all microfilariae (Mf) ingested by mosquitoes survive and develop into infective L3 larvae. To directly measure transmission potential, the presence of L3 in the vector must be evaluated.

Until recently, L3 detection in mosquitoes has only been possible by dissection of individual mosquitoes. Dissection is not practical nor is it sensitive enough for detecting and measuring mosquito infection and infectivity when rates are very low following MDA. Although many diagnostic tools are available to measure LF in communities, the lack of an efficient method of specifically detecting *W. bancrofti* L3 in vectors hampers the ability of elimination programs to evaluate transmission.

A molecular test specific to *W. bancrofti* infective mosquitoes would be useful for evaluating the success of GPELF by monitoring the decline of transmission risk following MDA. It would also provide important information regarding possible endpoints for MDA and for detecting resurgent infection following cessation of MDA. An L3 assay would also be useful for improving understanding of LF parasite/vector complexes in many geographic regions. For example, the mosquito species that transmit LF are not clearly defined in many parts of Africa [15]. Thus, an L3 assay could be used to identify or confirm species responsible for LF transmission. PCR tests cannot be used in this way because parasite DNA can be detected in non-vector mosquito species for weeks after they ingest filarial Mf [16].

In this work, we identified *W. bancrofti* L3-activated gene targets (mRNA transcripts first expressed in infective stage larvae) and developed a WbL3-detection assay that can determine the presence or absence of the infective stage of *W. bancrofti* in mosquito vectors using reverse transcriptase polymerase chain reaction (RT-PCR). This multiplex assay can simultaneously detect ‘any stage’ of the parasite in pools of mosquitoes yielding both infection and transmission potential data from the same samples. Most importantly, this new WbL3-diagnostic tool has the potential to improve our understanding of the impact of MDA on LF transmission.

## Methods

### Ethics Statement

This study was conducted according to the principles expressed in the Declaration of Helsinki. The study was approved by the Institutional Review Board of Ain Shams University and Washington University School of Medicine (Reference No. 99-0483). All patients provided written informed consent for the collection of samples and subsequent analysis. All microfilaremic volunteers were treated with DEC (6 mg/kg) plus Albendazole (400 mg) after mosquito feeding.

### Finding a *W. bancrofti* L3-Activated Gene

#### Search strategy and selection criteria for L3-diagnostic gene candidates

Cuticle genes were the focus of this analysis because cuticle collagens are known to be heterochronically expressed in nematode worms [17] and because our previous work identified a cuticle collagen as L3-activated in the closely related filarial parasite, *Brugia malayi* [18]. A list of *B. malayi* collagen genes was generated by searching gene clusters in the *B. malayi* Gene Index Database (http://compbio.dfci.harvard.edu/tgi/cgi-bin/tgi/gimain.pl?gudb=b_malayi) using the keyword “collagen”. Each *B. malayi* cuticle collagen cluster sequence was then compared against filarial sequences in dbEST to identify *W. bancrofti* orthologues using the NCBI BLASTN search tool [19]. Potential diagnostic gene targets identified using bioinformatics were excluded if pre-L3 expression was noted in any filarial species.

Additionally, potential targets for a WbL3-activated gene were identified using the free-living *Caenorhabditis elegans* dauer larvae as a model because the dauer stage is thought to be analogous to the L3 stage of parasitic nematodes [20]. Dauer-specific genes were identified using the *C. elegans* Wormbase Expression Pattern Search Tool (http://www.wormbase.org/db/searches/expr_search) with search criteria selecting genes that were ‘expressed in’ dauer larva and ‘not expressed in’ the embryo, postembryonic, all stage, L1 larva, or L2 larva cDNA libraries of *C. elegans*. This search identified a non-collagen cuticle component, cuticlin, as being dauer-specific. This *cut-1* sequence (Accession # C59636.2) was searched against the non-redundant NCBI protein database using BLASTX (Basic Local Alignment Search Tool) to search a translated nucleotide query against an amino acid/protein database [21]. The matching cuticlin protein sequence from the related filarial parasite that causes dog heartworm, *Dirofilaria immitis* (Accession # AAQ04694.1), was identified and then searched against filarial sequences in dbEST using the tBLASTN search tool (searches a translated nucleotide database using a protein query). This identified *W. bancrofti* cuticlin sequences that were aligned into three clusters using the SeqMan Program of the Lasergene Suite (DNAStar, Inc.) and labeled *cut-1.0*, *cut-1.1*, and *cut-1.2*.

#### Identification of intron-exon boundaries

The list of candidate *W. bancrofti* sequences was searched against the database of *B. malayi* genomic sequences (http://blast.jcvi.org/er-blast/index.cgi?project=bma1, BMA_1 scaffold database). Intron-exon boundaries were identified by comparing the *W. bancrofti* cDNA sequences with the corresponding *B. malayi* genomic sequences using the NCBI SPIDEY algorithm (www.ncbi.nlm.nih.gov/spidey) (an mRNA-to-genomic DNA sequence comparison tool). Gene candidates were excluded from further consideration if no corresponding genomic sequence was available to identify intron-exon boundaries necessary for primer/probe design to prevent detection of genomic DNA (gDNA).

#### Primer and probe design

Probes and primers were designed using the standard Taqman assay design parameters of the Primer Express program version 2.0 (Applied Biosystems, Inc., Foster City, CA). In addition, the following criteria were used: 1) the primers for the conventional RT-PCR assay were designed to span an exon-exon boundary to prevent the amplification of gDNA, 2) the probes for real-time RT-PCR were designed to span an exon-exon boundary to eliminate detection of gDNA, 3) whenever possible primers were designed to include one or more single nucleotide polymorphisms (SNPs) between *Brugia* and *Wuchereria* to enable species-specific amplification. All primers were synthesized by IDT, Inc. (Integrated DNA Technologies, Coralville, IA). Probes were synthesized either by Applied Biosystems, Inc or IDT, Inc. All primer and probe sequences used in this study are listed in Table 1.

**Table 1:** Primer and probe sequences.

Primer/Probe #	Gene or TC Identifier	Direction	Sequence 5′→3′
1054	TC7799*tph-1*	F	AAGGACGGCAAGTAGTAAGGA
1059	TC7799*tph-1*	R	AACAATTCATTTCTTGTAGC
1248	TC7799*tph-1*	P	VIC-ATCGGTGAGCGTATGGCCGAAGG- TAMRA
1251	TC7799*tph-1*	R	CTACTACAGCTACTTGTCCCTCACCTT
1252	TC7799*tph-1*	F	GACCGATTTAAACAGTTGCAGTTC
1765	TC7872	F	GGCCTATGTTGTACATGTCAACGT
1766	TC7872	R	ATATCACCAATAACACCATCGATACC
1767	TC7872	P	6FAM-ACCGGGACCTGATGGCGTGGAC-IB
1771	TC8016	F	GACGACCGGGACCTGTTG
1772	TC8016	R	TGGTGGACAATGGTCACAACTT
1773	TC8016	P	6FAM-CTGGAGAACCTGGTGCAC-IB
1774	TC8065	F	TGAAAGCCTTGTTTTACGAGCTAA
1775	TC8065	R	ACCTTTTGGACATCGGTCAGA
1776	TC8065	P	6FAM-TTTCCAGCTCATTGCCAATGTAG-IB
1778	TC8225	R	GCATGGACAGTAATGAGCATCTG
1779	TC8225	P	6FAM- AATGGCAAAACTGGAACGCCAGGTAGCA- IB
1787	TC7803	F	GAAATCCCGGAAGAGCCG
1789	TC7803	P	6FAM-AGGAAATAGTGGCAGTGCT-IB
1795	TC7967	P	6FAM-AACACGACGTTAAGATTGAT-IB
1796	TC8225	F	GGTGAACAGGGACCACTTGGTAG
1846	TC7920	F	AGGTACTGCTGGAACACCTGGC
1848	TC7920	P	6FAM-ACCCGGAGAGCCTGGA-MGBNFQ
1852	TC7803	R	CCGTCAGCTCCTGGCTGT
1853	TC7967	R	AATGATGATGAAAACCAATGCACA
1887	TC7967	F	ACCTGGACAACCAGGAGAACG
1889	TC7920	R	GCTGCATCACCGCCTGGTAT
1899	*Wb cut-1.0*	P	6FAM-CGAAGACAATCAAGCTCTA- MGBNFQ
1900	*Wb cut-1.0*	F	CGTCGACGTGAGGACTGATATC
1901	*Wb cut-1.0*	R	CATTGGTTGTCCACCGAGG
1908	*Wb cut-1.1*	F	GTCCACTCCTGCTTTGTGGAC
1909	*Wb cut-1.1*	R	CCCATAAACATTGAGAAGCCG
1910	*Wb cut-1.1*	P	6FAM- ACGGTGGAAATTCTGAATGCAGTACAAAA CG-TAMRA
1911	*Wb cut-1.2*	F	CACACAAATTGAAGTTTCCGAAATT
1912	*Wb cut-1.2*	R	TTATGATAAACCGGTTGTCCAATG
1913	*Wb cut-1.2*	P	6FAM- TGCCTGTATGTCGATATGAGATTCTTGAT GGTG-TAMRA
1936	TC7859*Wb-col-2*	F	6FAM-CAACCTCATATTGAATGGTGTT- MGBNFQ
1938	*Wb-cut*-1.2	F	AAATGAAGAGTTTACCTCAT
1939	*Wb-cut*-1.2	R	CCGGTTATTGACATACATA

*Vasuki, et al. (ref # 26).

Gene Identifier = TIGR Cluster Number (TC) available at http://compbio.dfci.harvard.edu/tgi/cgi-bin/tgi/gimain.pl?gudb=b_malayi.

F = Forward, R = Reverse, P = Probe (Forward orientation).

6FAM & VIC = Fluorescent Molecule on 5′ end of probe, TAMRA = Fluorescent Quencher (Applied Biosystems, Inc.), MGBNFQ = Minor Groove Binding Non-Fluorescent Quencher (Applied Biosystems, Inc.), IB = Iowa Black (Integrated DNA Technologies, Inc., Coralville, IA, USA).

#### Infected mosquito time course

A developmental time-course covering the extrinsic incubation period of *W. bancrofti* in mosquitoes was used to examine expression profiles and to identify the precise timing of the first expression of target gene candidates. *W. bancrofti* infected *Culex pipiens* mosquitoes were collected according to previously published protocols [22]. Briefly, *Cx. pipiens* larvae were collected and reared in an insectary at 27°C±2°C at 80% relative humidity. Emerging females were maintained on a 10% sugar solution. Four to five day old *Cx. pipiens* females were starved for 24 hours and then exposed to informed and consenting microfilaremic volunteers at night for approximately 30 minutes. The volunteers used for this study had high Mf counts, exceeding 400 Mf/mL blood. Mosquitoes were collected daily for up to 13 days post blood meal (PBM) and immediately preserved in RNAlater solution. Twenty mosquitoes were dissected on alternate days to determine infection rates and to monitor the development of parasites in the mosquitoes across the time-course.

#### Time-course screening by qRT-PCR

Pools of mosquitoes were created for each daily time point using two infected mosquitoes plus eight unfed (uninfected) mosquitoes across the time-course for testing candidate gene transcripts. Mosquito plus parasite RNA was extracted using a phenol/guanidine thiocyanate extraction procedure as previously described [19] except these RNA samples were not DNase treated. Four additional biological replicates of the RNA time-course were generated for additional testing using pools containing three or four infected mosquitoes combined with uninfected mosquitoes.

qRT-PCR testing was performed across the time-course for each gene using gene-specific primers and 6FAM labeled probes with the TaqMan OneStep RT-PCR kit (Applied Biosystems, Inc., Foster City, CA) according to manufacturer's instructions in 25 µl volume reactions. The Absolute Quantification module of the Sequence Detection System Program version 1.3 was used on the Applied Biosystems 7300 Real-Time PCR System; all samples were run in duplicate or triplicate along with gDNA and negative PCR controls. The cycling conditions were 50°C for 30 min, 95°C for 10 min, followed by 40 cycles of 95°C for 15 sec and 60°C for 1 min.

#### Selection of a diagnostic target

L3-activated genes were confirmed by testing in five replicate samples for each time-point in the time-course. The diagnostic target for the assay was selected based on the following criteria: 1) the target produced a signal from mosquito time points with L3 stage parasites but not from time points with only Mf, L1, or L2 stage parasites, 2) expression of the target was sufficient for detection, 3) no false positives detected with filarial gDNA, 4) a PCR reaction efficiency between 90–110%, 5) specificity for the filarial parasite species targeted, and 6) sufficient sensitivity to detect one infective mosquito in a pool of mosquitoes. After criteria 1–3 were satisfied, the target gene was tested for species-specificity and PCR efficiency. The L3-diagnostic RT-PCR assay was then combined with the constitutively expressed *tph-1* target (Accession # CD374712.1) for simultaneous detection of infective stage parasites and ‘any-stage’ parasites, and this multiplex assay was tested for sensitivity.

### Designing and Optimizing the WbL3-Detection Assay

#### Conventional multiplex RT-PCR WbL3-detection assay

Conventional RT-PCR multiplex reactions were performed using standard conditions of the OneStep RT-PCR kit (Qiagen, Inc.) with 600 nM of each primer (*cut-1.2* forward primer #1938 and reverse primer #1939, and *tph-1* forward primer #1054 and reverse primer #1059, Table 1) and 1 µl RNA template in a 25 µl total volume. The thermal cycling conditions used were 50°C for 30 min, 95°C for 15 min, and 57°C for 5 min, followed by 40 cycles of 72°C for 90 sec, 94°C for 45 sec, 57°C for 45 sec, and a final 10 min extension step at 72°C. PCR products (8 µl) were separated by electrophoresis on a 3% agarose gel, detected by ethidium bromide staining, and visualized with ultraviolet light.

#### Real time multiplex RT-PCR WbL3-detection assay

The real-time *cut-1.2* L3-detection assay was multiplexed with the *tph-1* ‘any-stage’ gene. Applied Biosystems, Inc. synthesized TaqMan probes with a different fluorophore on the 5-prime end for each target and a TAMRA quencher molecule on the 3-prime end (Table 1, #1913 *cut-1.2* and #1248 *tph-1*). Primer and probe optimizations (including primer-limiting experiments) were performed for the multiplex reaction according to the Applied Biosystems, Inc. standard protocols using the TaqMan One-step RT-PCR Master mix and the Multiscribe reverse transcriptase enzyme with RNase inhibitor. All reactions were done in a 25 µl total volume with 2 µl of template RNA using a 50°C RT step. The optimized conditions for the *W. bancrofti* multiplex qRT-PCR assay were 12.5 µl TaqMan OneStep RT-PCR Master Mix (Applied Biosystems, Inc.), 0.625 µl 40× Multiscribe/RNase inhibitor, and 160 nM of each probe (#1913 *cut-1.2* and #1248 *tph-1*), 900 nM of each *cut-1.2* primer (#1912 and #1913), 50 nM forward *tph-1* primer (#1252) and 100 nM reverse *tph-1* primer (#1251) with 2 µl of template RNA. The cycling conditions were 50°C for 30 min, 95°C for 10 min, followed by 40 cycles of 95°C for 15 sec and 60°C for 1 min. The optimized RNA extraction and real time multiplex RT-PCR detection of *cut-1.2* and *tph-1* is detailed in Protocol S1.

#### Assay efficiency

The efficiency of the qRT-PCR assay was calculated using the slope of a 5-log dilution standard curve and the Stratagene, Inc. “qPCR slope to efficiency calculator” (http://www.stratagene.com/techtoolbox/calc/qpcr_slope_eff.aspx).

#### Specificity testing

Aedes aegypti mosquitoes were fed on infected blood with the related filarial parasites B. pahangi, B. malayi, and D. immitis and maintained in an insectary under standard conditions for times required for the development of L3. Mosquitoes were collected on day 11 PBM for B. pahangi, day 14 PBM for B. malayi, day 16 PBM for D. immitis and preserved in RNAlater. W. bancrofti infected mosquitoes collected on day 16 PBM were used for specificity testing. RNA was extracted from three biological replicates for each mosquito/parasite combination from pools comprised of five mosquitoes fed on infected blood combined with five uninfected mosquitoes. Pools of ten uninfected mosquitoes were also tested. All RNA extracts were tested in triplicate using the multiplex qRT-PCR diagnostic assays described above.

#### Sensitivity testing

The sensitivity of the WbL3-detection assay was assessed by mixing one potentially infective mosquito (day 16 PBM) with unfed control mosquitoes in pool sizes of 10, 15, 20, 25 and 30 mosquitoes. RNA extraction and multiplex qRT-PCR were performed as described on two biological replicates for each condition.

## Results

### Identification of a *W. bancrofti* L3-Activated Gene

#### Developmental profile of *W. bancrofti* in *Cx. pipiens*

The dissection results indicated a slightly slower initial rate of development for the L1 and L2 stages compared with previous studies [23],[24]. However, the first L3 was detected by dissection on day 8 PBM in agreement with the previous studies. Thus, day 0–7 PBM mosquitoes contained pre-L3 vector stages and day 8 PBM was the earliest time point when L3 larvae were observed; by day 12 PBM all mosquitoes that contained parasite had L3 stage larvae (Table 2). The overall infection rate was 62%, ranging from 50% to 70% for any one time-point. Infectivity rates on days 8, 10 and 12 PBM were 5%, 20% and 60%, respectively. These results indicated that we needed to identify a gene with no expression prior to day 8 PBM.

**Table 2:** Dissection of *W. bancrofti* infected *Cx. pipiens*.

Time Point #dPBM	# Mosq. Dissected	# Mosq. with parasite (%)	Expected stage*	Stages detected	Mean # parasite per mosq. (range)	# Mosq. with L3 (%)	Mean # L3 per mosq. (range)
2	20	13 (65)	L1	Mf	3 (1–11)	0 (0)	-
4	20	10 (50)	L1	L1	3 (1–10)	0 (0)	-
6	21	14 (67)	L2	L1	2 (1–6)	0 (0)	-
8	20	12 (60)	L2/L3	L1/L2/L3	10 (1–45)	1 (5)	1
10	20	14 (70)	L3	L1/L2/L3	2 (1–3)	4 (20)	1.3 (1–2)
12	20	12 (60)	L3	L3	4 (1–14)	12 (60)	4 (1–14)
Total	121	75 (62)	L1/L2/L3	Mf/L1/L2/L3	4.3 (1–45)	17 (14)	3.4 (1–14)

*Expected stage of development based on published studies.

dPBM = number of days post blood meal (after mosquitoes were fed on infected blood).

#### RNA extraction from *W. bancrofti* infected mosquitoes

High quality RNA was extracted from pools of infected mosquitoes collected daily for 13 days post-feeding on microfilaremic blood and preserved in RNAlater. The RNA yield of the time-course samples ranged from 13.8–90.1 µg, with a median yield of 56 µg and a mean of 57 µg. The ratio of absorbance readings between 260 and 280 nanometer wavelengths was in the range of 1.9–2.1 indicating that high purity RNA was extracted from all samples.

#### Selection and expression profile testing of candidate genes

Eleven cuticle-related genes were selected for expression profile screening through bioinformatics searches (Table 3). Eight *W. bancrofti* ESTs were orthologues of *B. malayi* cuticle collagen genes and three were homologues of the *C. elegans* dauer-specific *cut-1* cuticlin gene represented by six ESTs that clustered into three distinct *W. bancrofti* cuticlin genes. Of the eleven *W. bancrofti* cuticle genes screened for L3-activation, six collagens were clearly expressed in the pre-L3 stages of the parasite (Table 3). One collagen (Accession # CK726187) expressed in day 10 PBM mosquitoes was also detected at low levels inconsistently in day 6, 7 and 8 PBM samples. Due to these inconsistent results this collagen gene was dropped from further consideration as a diagnostic target. One cuticlin gene, *cut-1.1*, was not detected at any time point perhaps due to very low expression levels or problems with the primers/probe and was dropped from further study. Three of the candidate genes, one collagen (Accession # CK855471) and two cuticlins, labeled *cut-1.0* and *cut-1.2* (representative Accession # CK950096 and CK854857, respectively) were identified as being L3-activated in *W. bancrofti* (Table 3). In the biological replicate time points tested, the earliest detection of the *cut-1.0* gene was day 8 PBM, and the collagen and *cut-1.2* were first expressed on day 9 PBM in *W. bancrofti*-infected mosquitoes. These three genes were then examined in detail to determine whether they met the criteria for development of an L3-detection assay.

**Table 3:** *W. bancrofti* Cuticle Genes Evaluated for Expression Onset.

*B. malayi* Gene Index Identifier	(*W. bancrofti*) EST Representative Genbank Accn #	Primers/(Probe)	Earliest Time Point of Gene Expression Detected
*Wb cut-1.1*cuticlin	CK854973	1908–1909 (1910)	undetected
TC7920collagen	CK854756	1846–1889 (1848)	5 dPBML2
TC7967collagen	CK850692	1887–1853 (1795)	5 dPBML2
TC8016collagen	CK850453	1771–1772 (1773)	6 dPBML2
TC7859*Bm-col-2*	CK855340	1751–1753;1756–1757 (1936)	6 dPBML1/L2
TC8065collagen	CK854684	1774–1775 (1776)	7 dPBML2
TC7872collagen	CK850687	1765–1766 (1767)	8 dPBML2/L3
TC8225collagen	CK726187	1796–1778 (1779)	10 dPBM*WbL3
***Wb cut-1.0*** **cuticlin**	**CK850096** **CK854700** **CK850637**	**1900–1901 (1899)**	**8 dPBMWbL3**
**TC7803collagen**	**CK855471**	**1787–1852 (1789)**	**9 dPBMWbL3**
***Wb cut-1.2*** **cuticlin**	**CK854857AF125580**	**1911–1912 (1913)1938–1939**	**9 dPBMWbL3**

Bm Gene Index Identifier = *B. malayi* TIGR Cluster Number (TC) available at http://compbio.dfci.harvard.edu/tgi/cgi-bin/tgi/gimain.pl?gudb=b_malayi.

Wb = *W. bancrofti*, *cut* = cuticlin, WbL3 = *W. bancrofti* L3 stage larvae, dPBM = days post blood meal (collection point after mosquitoes were fed on infected blood), *(ambiguous weak detection beginning at 6 dPBM).

#### Selection of a diagnostic target

The *W. bancrofti* L3-activated collagen gene (CK855471) met the assay criteria for expression level, no detection of gDNA, and PCR efficiency. Although our primer/probe set did not detect the transcript in *B. malayi* or *D. immitis* L3-infected mosquitoes; it was detected in *B. pahangi* L3. Further evaluation of the expression profile in a 14-day *B. pahangi* infected mosquito time-course indicated that this collagen was first detected at day 5 PBM, most likely corresponding to *B. pahangi* L2 expression. Therefore, this collagen gene was considered to be unsuitable as a *W. bancrofti* L3-diagnostic target. Both the *cut-1.0* and *cut-1.2* gene assays gave excellent results with regard to the diagnostic criteria outlined above including no detection in the three other filarial species tested. The *W. bancrofti cut-1.2* cuticlin gene was selected as the diagnostic target for the L3-detection assay, therefore, only *cut-1.2* assay results are shown.

### Designing and Optimizing the WbL3-Detection Assay

#### Real time multiplex RT-PCR WbL3-detection assay

**Figure 1:**
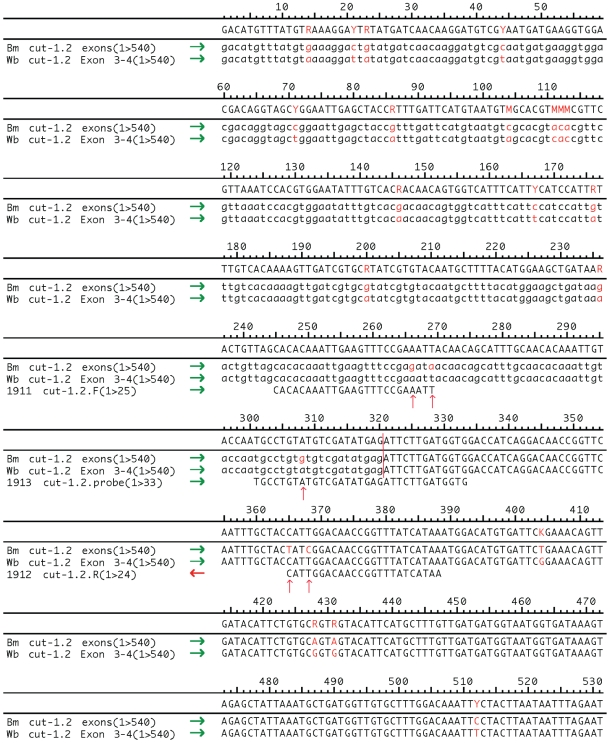
Primer and probe alignment with *cut-1.2* sequences of *W. bancrofti* and *B. malayi*. A portion of the *cut-1.2* sequences from *B. malayi* and *W. bancrofti* have been aligned with the primers and probes designed for the qRT-PCR *cut-1.2* detection assay. The nucleotides in red (red arrows) represent single nucleotide polymorphisms between the *Brugia* and *Wuchereria* transcripts that provide the specificity of the target. The exons are differentiated in the *W. bancrofti* sequence by lower and upper case letters (vertical red bar). The probe spans the exon-exon boundary to prevent detection of any contaminating genomic DNA.

The *W. bancrofti cut-1.2* expressed sequence tag (EST) contained six exons identified by SPIDEY alignment with the corresponding *B. malayi* genome sequence (TIGR_Assembly_12864, BMA_1 scaffold database). Primers (#1911 and #1912) amplified a 144 base pair product. The forward and reverse primers each have two SNP sites as compared with the *B. malayi* cuticlin sequence. One nucleotide difference between the two species is on the 3′ end of each primer (Figure 1) making these primers specific for *W. bancrofti*. The probe (#1913) also contained an internal SNP and spanned the boundary between exons three and four eliminating detection of gDNA. The efficiency of this reaction was 101.87% based on the slope (−3.277875) of a 5-log dilution standard curve.

The earliest time point of expression detected for *cut-1.2* was day 9 PBM in two of five time-course sets, but the Ct values were quite high (>38) indicating only a low level of expression. In two other time-course sets expression was first detected on day 10 PBM (Ct values of 38.2 and 38.8) and in the fifth time-course replicate the first time point of expression was day 13 PBM with a Ct value of 30 (day 11 and 12 were not represented in that time-course set) (Table 4). These results correspond nicely to the peak L3 development period in the vector (Table 2). In nearly all cases the Ct values for the later time points (days 13 and 16 PBM) were significantly lower than those of the earlier time points (stronger expression signals). This is probably because more L3 were present in mosquitoes collected at the later time points, although it is also possible that expression of this gene increases in the days following the molt to L3.

**Table 4:** *W. bancrofti cut-1.2* Expression Timeline.

Mosquito Time Point (#dPBM)	Expected stage of parasite development	*Wb cut-1.2* Ct value for Each of 5 Biological Replicates Indicating the Stage of Expression				
		a (2∶10)*	b (3∶8)*	c (3∶10)*	d (4∶10)*	e (3∶10)*
0	Mf	-	-	-	-	-
1	Mf	-	NPR	-	-	-
2	Mf/L1	-	-	-	-	-
3	L1	-	-	-	-	-
4	L1	-	-	-	-	-
5	L1/L2	-	-	-	-	-
6	L2	-	-	-	-	-
7	L2	-	-	-	-	-
8	L2/L3	-	-	-	-	-
9	L2/L3	NPR	38.15	-	38.83	-
10	L3	-	38.14	38.27	35.99	37.20
11	L3	nt	nt	34.77	37.64	-
12	L3	nt	nt	-	-	-
13	L3	30.12	25.98	22.63	nt	37.94
16	L3	nt	nt	24.74	25.72	- **

# dPBM = number of days post infected blood meal, *ratio of infected mosquitoes to total pool size, ** only 1 infected mosquito in this 16 dPBM sample. NPR indicates no parasite RNA (no *tph-1* detection) in that mosquito pool, nt = not tested due to insufficient sample remaining, “-” indicates no *cut-1.2* RNA detected in that sample, Ct value = cycle threshold value (product amplification detected to cross the threshold) where a lower Ct value indicates a higher level of expression.

#### Sensitivity testing

One *W. bancrofti* infective mosquito (day 16 PBM) was detected with the *cut-1.2* assay in pools of up to 30 mosquitoes with Ct values ranging from 22–33 (Table 5).

**Table 5:** Sensitivity of *W. bancrofti cut-1.2* L3-Detection Assay.

Sample ID	# infective mosq (16 dPBM)	Total Pool Size	RNA Yield (µg)	Mean Ct (*cut-1.2*)
WbS-1.10A	1	10	80	27.825
WbS-1.10B	1	10	140	27.73
WbS-1.15A	1	15	157	25.405
WbS-1.15B	1	15	158	25.62
WbS-1.20A	1	20	196	24.48
WbS-1.20B	1	20	204	27.695
WbS-1.25A	1	25	241	low *tph-1*
WbS-1.25B	1	25	232	no *tph-1*
WbS-1.30A	1	30	256	33.43
WbS-1.30B	1	30	299	24.955
WbS-3.10A	3	10	104	22.12
Un-Cp	0	10	109	0

dPBM = days post infected blood meal; Un-Cp = unfed *Cx. pipiens*.

Samples with low or no *tph-1* ‘any-stage’ control gene detection indicated little or no parasite RNA in that sample.

#### Specificity testing

The *cut-1.2* transcript was not detected in mosquitoes with *D. immitis*, *B. malayi*, or *B. pahangi* and it was also not detected in unexposed *Cx. pipiens* or *Ae. aegypti* mosquitoes. Positive results were only observed in samples containing *W. bancrofti* L3.

#### Conventional multiplex RT-PCR assay

**Figure 2:**
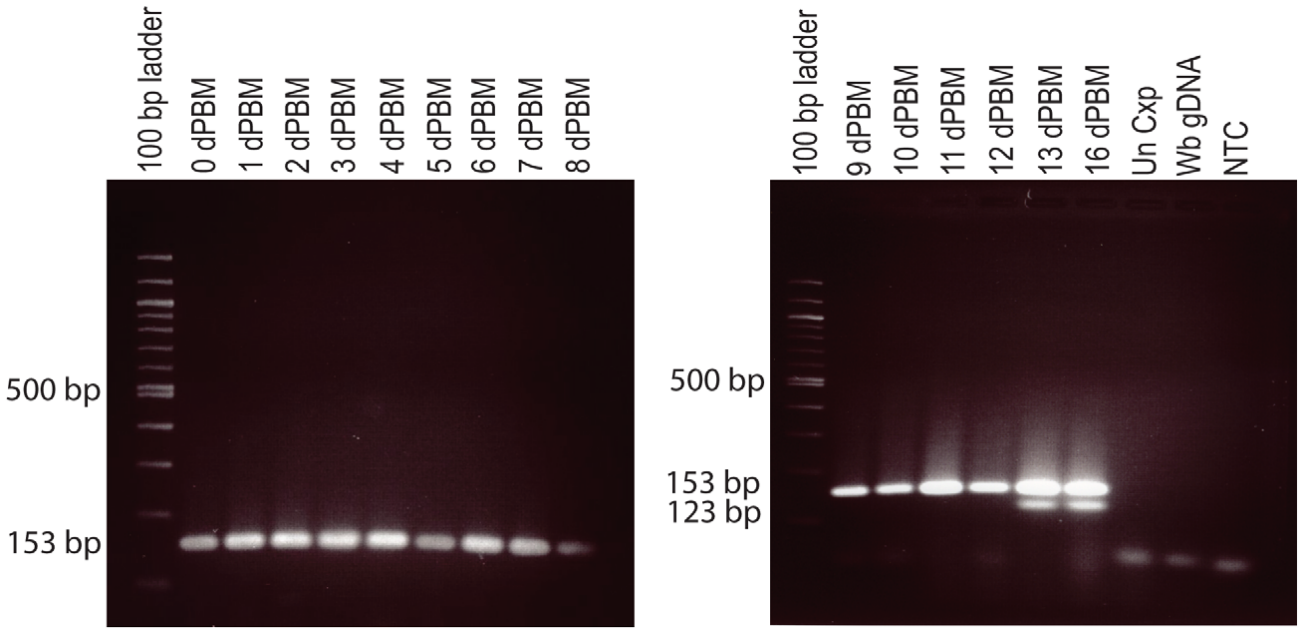
Conventional RT-PCR detection of *W. bancrofti tph-1* and *cut-1.2* in a mosquito time-course. Time-course set C illustrates no amplification of the L3-activated *cut-1.2* transcript (123 bp) in time-points prior to L3 development, while the control *tph-1* transcript (153 bp) is detected in all time-points indicating that parasite RNA was present. Panel A shows mosquitoes collected from 0–8 days dPBM and panel B shows mosquitoes collected 9–13, and 16 dPBM. dPBM = the number of days post blood meal, Un Cxp = Unfed *Cx. pipiens* mosquitoes, Wb gDNA = *W. bancrofti* genomic DNA, NTC = No template control.

**Figure 3:**
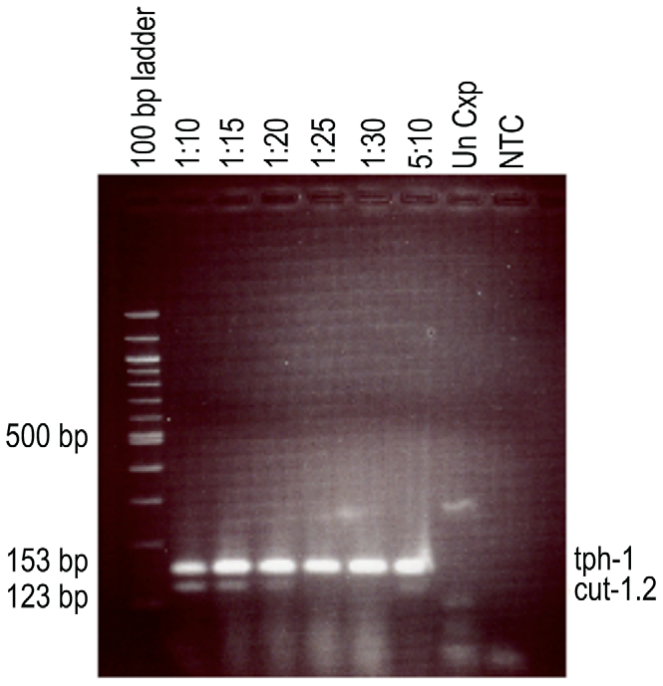
Sensitivity testing of the *W. bancrofti* multiplex L3-detection assay by conventional RT-PCR. The *tph-1* transcript (‘any-stage’ detection) is detected in all samples, while the *cut-1.2* transcript (L3-detection) is only detected in samples of pool size up to 20 mosquitoes. The L3-detection sensitivity limit by conventional RT-PCR is one infective mosquito in a pool of 20 mosquitoes. 1∶10 = one bloodfed mosquito (day 16 post blood meal) in a pool of 10 mosquitoes, 1∶15 = one bloodfed mosquito in a pool of 15 mosquitoes, etc., 5∶10 = 5 bloodfed mosquitoes in a pool of 10 mosquitoes. Un Cxp = Unfed *Cx. pipiens*, NTC = no template control.

*Wb-cut-1.2* primers for conventional RT-PCR amplified a 123 base pair (bp) product from WbL3 RNA, while primers for *tph-1* amplified a 153 bp fragment from all stages of the parasite. The conventional RT-PCR assay primers were designed to cross an exon junction and they did not amplify gDNA. The size difference between the *tph-1* and *cut-1.2* amplification products allowed them to be differentiated on a 3% agarose gel. Figure 2 shows an example of the expression pattern of *tph-1* and *cut-1.2* in infected mosquitoes that were tested at different time points using conventional RT-PCR. *tph-1* expression was detected in all infected mosquito pools, whereas *cut-1.2* expression was not detected until day 13 PBM when L3 are present. The conventional *cut-1.2* RT-PCR primer set was specific for *W. bancrofti* samples; no expression was detected in mosquitoes with *D. immitis*, *B. malayi*, or *B. pahangi* L3 or in uninfected control mosquitoes (data not shown). Sensitivity testing indicated that *tph-1* expression was detected in all pool sizes tested (one infected mosquito in 10, 15, 20, 25, and 30 mosquitoes). The *cut-1.2* L3-detection target was detected in pools of 10, 15 and 20 mosquitoes (Figure 3).

## Discussion

An assay to detect filarial L3 in pools of mosquitoes requires 1) a ‘field-friendly’ method of collecting mosquitoes that preserves parasite RNA, 2) an effective RNA extraction method that isolates parasite RNA along with mosquito RNA, 3) identification of an L3-activated gene to ensure only infective stage parasites are detected, and 4) sensitive and species-specific detection of the L3-activated gene. The first two requirements, preservation and extraction of parasite RNA in mosquitoes, were successfully accomplished during our previous work on *Brugia* L3-detection [19]. However, we were unable to identify a *W. bancrofti* orthologue of the *B. malayi* L3-activated diagnostic target (Accession # AA585578). This necessitated a search for a different L3-activated gene in *W. bancrofti*.

Our strategy used bioinformatics to identify cuticle genes. Cuticle genes are known to have a heterochronic expression pattern in free-living nematode worms [17],[25]. In addition, a cuticle collagen gene was identified in our previous work as being L3-activated in *B. malayi* [18]. In this study we identified eight *W. bancrofti* collagens and three cuticlins and examined their expression profiles in detail using RNA isolated from mosquitoes at daily time points after feeding on infected blood. One of the eight collagen genes identified in this search (Accession # CK855340) was a gene that was previously identified by Vasuki et al [26] (Accession # EU370160) as being a *W. bancrofti* L3-diagnostic gene (*col-2*). However, we found that *W. bancrofti col-2* was expressed on day 6 PBM, prior to the first appearance of L3 on day 8 PBM in mosquitoes reared for this study. Thus, this gene was not L3-activated. Of the eleven cuticle-related targets investigated in this study, we identified three *W. bancrofti* L3-activated genes including one collagen (Accession # CK855471) and two cuticlin genes (Accession # CK850637 and # AF125580).

Specificity of the primer/probe sets for the L3-activated targets was evaluated using *B. malayi*, *B. pahangi*, and *D. immitis* infective mosquitoes due to availability of laboratory animal models for these species. There are other filarial parasites in endemic areas, but L3 stage material from these parasites was unavailable for testing. Nonetheless, given that the *Wuchereria* and *Brugia* species are very close evolutionary neighbors [27] it is highly unlikely that a gene from a more distantly-related species would be more similar in sequence at the nucleotide level. *W. bancrofti* specificity of both the *cut-1.0* and *cut-1.2* assays was confirmed, while the *W. bancrofti* L3-activated collagen transcript (CK855471) was detected in mosquitoes harboring *B. pahangi* L2 stage parasites making it unsuitable as a target for WbL3-detection in field samples. Although both cuticlins could be suitable targets for L3 detection, we selected *cut-1.2* for the WbL3-detection assay due to the slightly later time point of earliest detection (day 9PBM versus day 8PBM).

One infective mosquito was detected in a pool of up to 30 mosquitoes using the multiplex real-time RT-PCR L3-detection assay with *tph-1* and *cut-1.2*. As expected, the conventional assay is slightly less sensitive, detecting one infective mosquito in a pool of up to 20 mosquitoes. A potential limitation of this study is that the WbL3 assay was not tested at the level of single worm detection due to the difficulty in obtaining isolated *W. bancrofti* L3 parasites preserved for RNA extraction (fast-frozen in liquid nitrogen). It is not possible to preserve single worms in RNAlater solution because the high salt content does not allow the separation of the worm from the solution for RNA extraction. Thus, the sensitivity of the WbL3-detection assay can only be stated as per infective mosquito, not per L3 parasite.

With methods previously used for *B. malayi*, we multiplexed the WbL3-detection target with the constitutively expressed control gene *tph-1* to enable simultaneous ‘any-stage’ detection in a standard RT-PCR assay. This allows both xenomonitoring and transmission risk to be evaluated in one test. It is important to note that the *tph-1* assay detects an expression signal from both *Brugia* and *Wuchereria*, but not from the related zoonotic parasite *D. immitis*.

One consideration for the implementation of any new diagnostic technique is the practicality of using it as a monitoring or surveillance tool in the field. The storage of vectors in RNAlater eliminates any major limitations regarding mosquito collection. The mosquitoes can be stored for at least one day at ambient temperature and for several months to even years at −20°C or −80°C. Any laboratory that is already performing PCR would be able to use the conventional RT-PCR assays with no additional equipment investment. For the real-time assay, the investment of a real-time PCR instrument would be necessary in laboratories that do not already have such an instrument. The advantages to the real-time assay include a higher throughput level (reduced labor investment), increased sensitivity, as well as a reduction in potential contamination due to the elimination of post-PCR product handling. The real-time assay is a more cost efficient test and it is the preferred test to use if the equipment is available. Studies are currently underway to validate this new diagnostic tool for use in field-caught mosquitoes.

Over the past few decades much progress has been made in advancing diagnosis of LF but not in monitoring transmission. GPELF currently uses indirect human measures to evaluate the success of its primary goal, the interruption of transmission. An L3-detection assay provides a more direct measure of transmission risk and may be useful as a sensitive and non-invasive method for monitoring GPELF programs. This multiplex L3/‘any-stage’ detection assay could also be a non-invasive surveillance tool for early detection of LF resurgence following suspension of MDA by detecting both Mf in the community and potential transmission risk. L3 detection may also be useful for identifying mosquito species that are LF vectors in areas where this is not already known; non-vector mosquitoes should not harbor L3. Finally, this new tool may also be used to answer research questions such as the seasonality of transmission or the effect of MDA on transmission rates.

## Supporting Information

Protocol S1 WbL3-detection assay SOP. A detailed step-by-step protocol for the RNA extraction and real-time RT-PCR assay.
(0.04 MB DOC)

## References

[1] Michael E, Bundy DA (1997). Global mapping of lymphatic filariasis.. Parasitol Today.

[2] Durrheim DN, Wynd S, Liese B, Gyapong JO (2004). Editorial: Lymphatic filariasis endemicity–an indicator of poverty?. Trop Med Int Health.

[3] Ottesen EA, Hooper PJ, Bradley M, Biswas G (2008). The global programme to eliminate lymphatic filariasis: health impact after 8 years.. PLoS Negl Trop Dis.

[4] Ottesen EA (2000). The global programme to eliminate lymphatic filariasis.. Trop Med Int Health.

[5] Molyneux DH, Zagaria N (2002). Lymphatic filariasis elimination: progress in global programme development.. Ann Trop Med Parasitol.

[6] Weil GJ, Ramzy RM (2007). Diagnostic tools for filariasis elimination programs.. Trends Parasitol.

[7] Weil GJ, Kastens W, Susapu M, Laney SJ, Williams SA (2008). The impact of repeated rounds of mass drug administration with diethylcarbamazine plus albendazole on bancroftian filariasis in Papua New Guinea.. PLoS Negl Trop Dis.

[8] Ramzy RM, El Setouhy M, Helmy H, Ahmed ES, Abd Elaziz KM (2006). Effect of yearly mass drug administration with diethylcarbamazine and albendazole on bancroftian filariasis in Egypt: a comprehensive assessment.. Lancet.

[9] Williams SA, Laney SJ, Bierwert LA, Saunders LJ, Boakye DA (2002). Development and standardization of a rapid, PCR-based method for the detection of *Wuchereria bancrofti* in mosquitoes, for xenomonitoring the human prevalence of bancroftian filariasis.. Ann Trop Med Parasitol.

[10] Rao RU, Atkinson LJ, Ramzy RM, Helmy H, Farid HA (2006). A real-time PCR-based assay for detection of *Wuchereria bancrofti* DNA in blood and mosquitoes.. Am J Trop Med Hyg.

[11] Plichart C, Sechan Y, Davies N, Legrand AM (2006). PCR and dissection as tools to monitor filarial infection of *Aedes polynesiensis* mosquitoes in French Polynesia.. Filaria J.

[12] Plichart C, Laney SJ, Sechan Y, Davies N, Legrand A-M (2007). Correction: PCR and dissection as tools to monitor filarial infections of *Aedes polynesiensis* mosquitoes in French Polynesia.. Filaria J.

[13] Bockarie MJ (2007). Molecular xenomonitoring of lymphatic filariasis.. Am J Trop Med Hyg.

[14] Farid HA, Morsy ZS, Helmy H, Ramzy RM, El Setouhy M (2007). A critical appraisal of molecular xenomonitoring as a tool for assessing progress toward elimination of Lymphatic Filariasis.. Am J Trop Med Hyg.

[15] Goodman DS, Orelus JN, Roberts JM, Lammie PJ, Streit TG (2003). PCR and Mosquito dissection as tools to monitor filarial infection levels following mass treatment.. Filaria J.

[16] Fischer P, Erickson SM, Fischer K, Fuchs JF, Rao RU (2007). Persistence of *Brugia malayi* DNA in vector and non-vector mosquitoes: implications for xenomonitoring and transmission monitoring of lymphatic filariasis.. Am J Trop Med Hyg.

[17] Liu Z, Kirch S, Ambros V (1995). The *Caenorhabditis elegans* heterochronic gene pathway controls stage-specific transcription of collagen genes.. Development.

[18] Laney SJ, Buttaro CJ, Visconti S, Pilotte N, Ramzy RM (2008). A Reverse Transcriptase-PCR Assay for Detecting Filarial Infective Larvae in Mosquitoes.. PLoS Negl Trop Dis.

[19] Altschul SF MT, Schaffer AA, Zhang J, Zhang Z (1997). Gapped BLAST and PSI-BLAST: A new generation of protein database search programs.. Nucleic Acids Res.

[20] Burglin TR, Lobos E, Blaxter ML (1998). *Caenorhabditis elegans* as a model for parasitic nematodes.. Int J Parasitol.

[21] Altschul SF, Gish W, Miller W, Myers EW, Lipman DJ (1990). Basic local alignment search tool.. J Mol Biol.

[22] Farid HA, Hammad RE, Hassan MM, Ramzy RM, El Setouhy M (2005). Effects of combined diethylcarbamazine and albendazole treatment of bancroftian filariasis on parasite uptake and development in *Culex pipiens L*.. Am J Trop Med Hyg.

[23] Gad AM, Farid HA, Hammad RE, Hussein MA, Kaschef AH (1996). Host-parasite relationships of *Wuchereria bancrofti* and mosquito hosts, *Culex pipiens L*. and *Aedes caspius pallas*.. J Egypt Soc Parasitol.

[24] Gad AM, Hammad RE, Farid HA (1996). Uptake and development of *Wuchereria bancrofti* in *Culex pipiens L*. and *Aedes caspius pallas*.. J Egypt Soc Parasitol.

[25] Sebastiano MLF, Bazzicalupo P (1991). *cut-1* a *Caenorhabditis elegans* gene coding for a dauer-specific noncollagenous component of the cuticle.. Dev Biol.

[26] Vasuki V, Hoti SL, Patra KP (2008). RT-PCR assay for the detection of infective (L3) larvae of lymphatic filarial parasite, *Wuchereria bancrofti*, in vector mosquito *Culex quinquefasciatus*.. J Vector Borne Dis.

[27] Xie H, Bain O, Williams SA (1994). Molecular phylogenetic studies on *Brugia* filariae using Hha I repeat sequences.. Parasite.

