# Supplementary material for: Detection of Wuchereria bancrofti L3 Larvae in Mosquitoes: A Reverse Transcriptase PCR Assay Evaluating Infection and Infectivity
Source: PLoS Negl Trop Dis. 2010 Feb 16;4(2):e602. doi: 10.1371/journal.pntd.0000602 (PMC2821903; doi:10.1371/journal.pntd.0000602)
Supplement: Protocol S1 — WbL3-detection assay SOP. A detailed step-by-step protocol for the RNA extraction and real-time RT-PCR assay. (0.04 MB DOC) [file pntd.0000602.s001.doc]

**Standard Operating Procedure for the Multiplex qRT-PCR *W. bancrofti* L3 Diagnostic Assay**

**Purpose:** To efficiently extract RNA from pools of mosquitoes preserved in RNAlater for the detection of *W. bancrofti* infected and infective mosquitoes using real-time RT-PCR.

**Scope:** This RNA extraction procedure is for pools of 5 to 30 mosquitoes that have been preserved in RNAlater solution (Ambion, Inc. #7021) according to the manufacturer’s instructions.

**Materials:**

2 mL round-bottom tubes (e.g. USA Scientific catalog #1620-2700)

TRI Reagent® (Ambion, Inc. Catalog #AM9738)

1-bromo-3-chloropropane (BCP) (MRC, Cat. #BP 151)

100% isopropanol ACS grade or better

100% ethanol ACS grade or better

Nuclease-free water (e.g., Ambion Catalog #9914G)

glycogen (e.g., Ambion’s glycoblue catalog # AM9516)

High Salt Solution for precipitation (e.g. 0.8 M sodium citrate and 1.2 M NaCl)

heavy phase-lock gel tubes (optional, e.g. Fisher Scientific Catalog # E0032 005 152) RNA storage solution (optional, e.g., Ambion Catalog # 7000 or 7001)

TaqMan One–step RT-PCR Master Mix (Applied Biosystems, Inc. Part # 4309169)

RNase-free tips and tubes

MicroAmp Optical 96-well Reaction Plate with Barcode and Optical Adhesive Films

(Applied Biosystems, Inc. Part #4314320)

Primer and Probe sequences:

***tph-1***

#1252 (5’-GACCGATTTAAACAGTTGCAGTTC-3’)

#1251 (5’-CTACTACAGCTACTTGTCCCTCACCTT-3’)

#1248 (VIC-ATCGGTGAGCGTATGGCCGAAGG- TAMRA™)

**WbL3 *cut-1.2*:**

#1911 (5’-CACACAAATTGAAGTTTCCGAAATT-3’)

#1912 (5’-TTATGATAAACCGGTTGTCCAATG-3’)

#1913 (6FAM- TGCCTGTATGTCGATATGAGATTCTTGATGGTG-TAMRA™)

**Procedure:** All supplies and reagents should be for RNA use only! Make sure all tubes and tips are RNase/DNase free.  Wear a clean lab coat.  Change gloves frequently.  Clean entire area and pipettes with RNaseZap (Ambion #9780) before beginning extractions. Pre-clean BBs with RNase-Zap and rinse in RNase-free water.

NOTE: TRI reagent (Ambion, Inc. catalog # AM9738) Reagent is toxic. Wear Gloves and lab coat. Use of TRI Reagent should be according to proper safety procedures. Dispose of TRI Reagent contaminated waste according to approved waste disposal procedures.

Note: Using a 2 ml round-bottom tube is important to allow for enough empty space within the tube for the metal ball bearing (BB) to vibrate sufficiently.

1. Centrifuge tubes at maximum speed briefly.
2. Completely remove RNAlater solution and rinse mosquitoes with 1 mL RNase-free water to remove any residual salt. (This is important because the excess salt concentration of the RNAlater will inhibit the efficient extraction of the RNA.) Alternatively, with clean forceps transfer the mosquitoes to a new tube after blotting excess RNAlater on kimwipes.
3. Add 1 BB to the 2 mL round-bottom tube.
4. Add 990 μl TRI Reagent (Ambion catalog #AM9738) and 10 μl glycoblue (15 mg/ml) [Ambion #9515].
5. Vortex horizontally for 30 minutes on high speed (note: homogenized samples can be stored at -70^o^C for at least 1 month at this step).
6. Let sit for (at least) 5 min @ room temperature (RT) to complete dissociation of ribonucleoproteins.
7. Centrifuge @ 12,000 x g for 10 min @ 4^o^C.
8. Transfer supernatant to a heavy phase lock gel tube (Fisher Scientific, Inc. Cat. # FP2302830).
9. Add 100 μl BCP (1-bromo-3-chloropropane, Molecular Research Center, Inc. Cat # BP 151) per 1 ml TRI reagent.
10. Shake vigorously for 15 sec. (do not vortex).
11. Incubate at RT for 5 – 15 min.
12. Spin @ 12,000 x g (no more) for 15 min @ 4^o^C. (centrifugation at > 8^o^C may cause some DNA to partition in the aqueous phase).
13. Add 100 μl BCP, shake vigorously for 30 sec.
14. Incubate at RT for 5 min.
15. Centrifuge @ 12,000 x g (no more) for 10 min @ 4^o^C
16. Transfer aqueous phase (colorless top layer) to a new 1.5 mL *labeled* tube.
17. Replacement step to remove proteoglycan and polysaccharide contamination: for each 1 ml of TRI reagent, add 250 μl of isopropanol and 250 μl of a high salt precipitation solution (0.8M sodium citrate, 1.2 M NaCl).
18. Vortex 5-10 sec, and incubate at RT for 10 min.
19. Centrifuge @ 12,000 x g for 8 min @ 4-25^o^C.
20. Locate pellet & remove supernatant (can dump carefully if pellet is visible).
21. Wash with 1 ml of 75% EtOH.
22. Briefly vortex to mix (look for loosened pellet).
23. Centrifuge @ no more than 7,500 x g for 5 min @ 4-25 ^o^C (if pellet floats, spin again at 12,000 x g for 5 min).
24. Remove EtOH with flame-drawn pipet (do not dump), spin briefly and remove residual EtOH.
25. Let air dry for 5-10 min on ice (do not use speed vacuum concentrator or let it dry too long because this will greatly decrease the solubility of RNA pellet).
26. Resuspend in 100 μl RNA Storage Solution (Ambion, cat #7000, 7001) or Nuclease-free water (Ambion cat # 9914G) or nuclease free 0.1x TE buffer.
27. Use 1.5 μl of a 10-fold dilution of RNA for concentration and purity analysis with NanoDrop spectrophotometer. The purity reading, A_260_/A_280_, should be in the range of 1.8-2.2.
28. Store at 4 ^o^C for immediate analysis or at -70 ^o^C for long-term storage.
29. Proceed with *Wb cut-1.2*/*tph-1* multiplex qRT-PCR assay.

***Wbcut-1.2*/*tph-1* multiplex qRT-PCR assay** (25 μl reaction volume):

(Set up reactions on ice)

12.5 μl Taqman One-step RT-PCR master mix

0.625 μl 40x Multiscribe/RNase inhibitor

2.25 μl #1911 *Wb cut-1.2* Forward primer (10 μM), final [900 nM]

2.25 μl #1912 *Wb cut-1.2* Reverse primer (10 μM), final [900 nM]

0.125 μl #1252 *tph-1* forward primer (10 μM), final [50 nM]

0.250 μl #1251 *tph-1* reverse primer (10 μM), final [100 nM]

0.40 μl #1913 *Wb cut-1.2* FAM.Tamra probe (10 μM), final [160 nM]

0.40 μl #1248 *tph-1* VIC.TAMRA probe (10 μM), final [160 nM]

4.20 μl RNase-free ddH_2_O

23 μl

2 μl RNA template

25 μl Total Volume

**Thermal cycling parameters for ABI 7300 TaqMan system:**

RT-step:

30 min @ 50^o^C

PCR:

10 min 95^o^C

40 cycles of:

15 sec @ 95^o^C

1 min @ 60^o^C
